# Potential toxic elements in surface water of Mokosh Beel, Gazipur, Bangladesh: Ecological and human health risk assessment for recreational users

**DOI:** 10.1016/j.heliyon.2025.e42421

**Published:** 2025-01-31

**Authors:** Md. Shahriar Mahmud, M. Safiur Rahman, S.A. Dina, M. Rifat Nasher, Tasrina R. Choudhury, Bilkis A. Begum, Abdus Samad

**Affiliations:** a Department of Chemistry, Jagannath University, Dhaka, 1100, Bangladesh; b Water Quality Research Laboratory, Chemistry Division, Water & Air Research Cell (WARC), Atomic Energy Center, Bangladesh Atomic Energy Commission, Dhaka, 1000, Bangladesh; c Department of Geography and Environment, Jagannath University, Dhaka, 1100, Bangladesh

**Keywords:** Mokosh Beel, Industrial effluents, Potential toxic elements, Pollution assessment, Human health risk, Ecological risk

## Abstract

Mokosh Beel, a significant wetland in Bangladesh, is increasingly impacted by industrial effluents, leading to potential ecological and human health risks. This study evaluates the surface water quality of Mokosh Beel by analyzing both physicochemical parameters (pH, DO, EC, TDS, and salinity) and the concentrations of potential toxic elements (PTEs) (i.e., Mn, Cu, Ni, Pb, As, Cd, Co, Cr, Sb, and Zn). The findings reveal that most water quality parameters, except pH, exceeded local and international guidelines, indicating poor water quality. Among the PTEs, Mn (269.13 μg/L), Cr (33.20 μg/L), and Pb (71.47 μg/L) surpassed recommended safety limits. The spatial distribution analysis identified Mn and Pb as the primary pollutants based on the single-factor pollution index. The Nemerow pollution index indicated mild to moderate pollution, while the heavy metal pollution index (HPI) and heavy metal evaluation index (HEI) suggested a low degree of pollution at most sampling sites. Principal component analysis (PCA) and hierarchical cluster analysis (HCA) linked the majority of PTEs to industrial sources, particularly from tannery, leather, and paint industries. The potential ecological risk index (PERI) showed minimal ecological risk, but the hazard index (HI) indicated non-carcinogenic risks for children, although adults were not significantly affected. Carcinogenic risk assessments highlighted Pb and Cd as key contributors, with risks exceeding the critical threshold for both children and adults. This study underscores the urgency of addressing industrial pollution to safeguard both ecological health and human well-being, particularly for vulnerable populations like children. Policymakers must implement sustainable water management strategies to mitigate the ongoing contamination of Mokosh Beel.

## 1 Introduction

Pollution of surface water due to the release of industrial effluent is a serious concern because of its severe effect on water quality, agriculture, and human health [1,2]. Since diverse industries are present in an industrial area, effluent containing various pollutants is released into the water body, causing ecological imbalance in the aquatic ecosystem [3]. Although industrialization is important for the economic development of a country, unplanned industrialization and untreated industrial effluent are the most severe polluting agents of surface water [2]. In developing countries, industrial effluent is usually discharged into the nearby wetlands, rivers, canals, lakes, crop fields, etc. Without any pretreatment [4]. These wastewaters may contain various harmful pollutants, including potential toxic elements (PTEs). The direct discharge of wastewater into surface water bodies from industrial, mining, and urban activities in the vicinity are the major sources of PTEs in aquatic systems [5]. The release of irrigation water containing pesticides and fertilizers, and domestic wastewater through unlined channels also plays a significant role in the increase of PTEs in aquatic systems [6]. Furthermore, the runoff from industrial solid waste dumps and fly ash dumps of thermal power plants also contributes to trace PTEs contamination [7]. The presence of PTEs in aquatic environments can harm species diversity and ecosystems due to their persistence, accumulation, and toxicity. Ultimately, these toxic elements bioaccumulate in human consumers, posing significant health risks [8]. Human beings can be exposed to PTEs through direct ingestion and dermal contact with the water. Additionally, the bioaccumulation of toxic substances in aquatic organisms enters the human food chain, potentially leading to toxic effects. Chronic exposure to PTEs may cause fatal diseases such as Alzheimer's, multiple sclerosis, Parkinson's, and cancer [4]. The potential toxic elements include essential and nonessential metals such as arsenic (As), chromium (Cr), zinc (Zn), cadmium (Cd), copper (Cu), and lead (Pb). Toxic metals like Cd, Pb, and As are nonessential metals that exhibit extreme toxicity even at very low concentrations in the human body [9]. Although copper (Cu) and zinc (Zn) are considered essential and beneficial minerals for the human body, an overdose of these elements can be detrimental to the human body and cause various health-related problems.

This study focuses on Mokosh Beel, one of the largest perennial 67 wetlands in the Dhaka division of Bangladesh, covering approximately 1100 ha during the wet season but shrinking to only 40 ha in the winter. This wetland serves as an agricultural field during the winter season and a recreation area during the wet season. As an integral part of local livelihoods and culture, Mokosh Beel plays a crucial role in fisheries, nutrient retention, floodwater storage, groundwater recharge and discharge, and water transport [10]. However, the quality of surface water in the Mokosh Beel area is worsening due to the establishment of a number of industrial units surrounding this area. Industries located in nearby areas use Mokosh Beel and water bodies around as the disposal grounds for their untreated waste effluent [11]. It has been reported that there are two tannery factory units, one aluminum factory, and hundreds of knitting, dyeing, printing, and pharmaceutical industries located around this Beel [12,13]. Although government authorities have mandated the use of effluent treatment plants (ETPs) to prevent water pollution, many factory owners are reluctant to comply due to weak law enforcement and the high costs associated with running ETPs. As a result, large quantities of untreated wastewater are discharged into surrounding water bodies, which flow into Mokosh Beel [13]. The release of such harmful wastewater has gradually degraded the water quality in the Beel to the point where it is now unsuitable for daily human use [12,13].

Although the surface water of Mokosh Beel is severely polluted due to various anthropogenic activities, limited research has focused on the pollution assessment of surface water and its potential health impacts on local residents [[12], [13], [14]]. The available literature has only focused on a few physicochemical parameters and the distribution of certain heavy metals. We are unaware of any study that discusses source identification, ecological risk, and health risk assessment related to PTEs in the surface water of Mokosh Beel. Therefore, the specific objectives of this study are to (i) investigate the physicochemical parameters such as pH, DO, EC, salinity, and TDS, and determine the concentrations of ten potential toxic metals (As, Sb, Cr, Mn, Ni, Co, Cu, Cd, Pb, and Zn) in the water of Mokosh Beel; (ii) assess the pollution levels using specific pollution indices (HPI, HEI, Pi, and Np); (iii) identify the sources of these ten potential toxic metals; and (iv) evaluate the potential human health risks posed by these toxic metals.

## 2 Materials and method

### 2.1 Sampling site

**Fig. 1:**
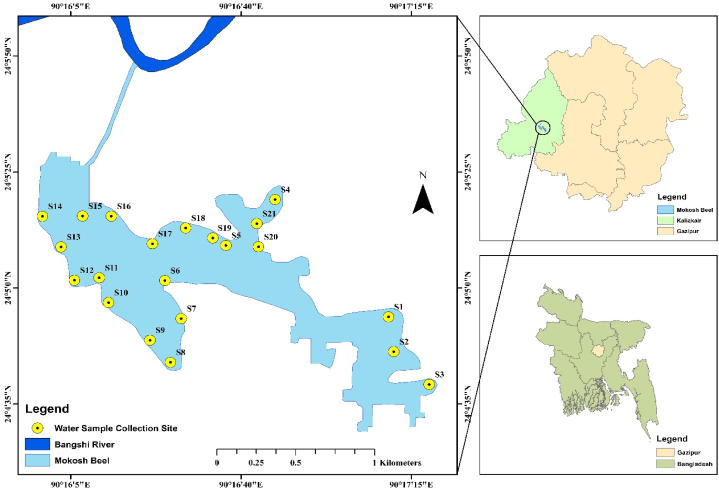
Sampling area map including sampling point of Mokosh Beel under Kaliakoir thana of Gazipur district, Bangladesh.

Samples were collected from Mokosh Beel (24°05′22.4″N, 90°16′30.9″E) in the Boroibari area under the Kaliakoir upazila of the Gazipur district. This Beel is situated approximately 25 km northeast of the capital city of Dhaka, Bangladesh, and to the west of the Turag River. The Turag-Bangshi site is mostly a low-lying floodplain of Bangladesh located just north of Dhaka. The Saturia-Solahati canal connects this Beel with the Turag River. Due to industrial effluent and the dumping of plastic waste, the water level of this Beel does not decrease much in the dry season. This situation makes this area unfit for cultivation even in the dry season. In the rainy season, water spills over the riverbanks through different canals (carrying the effluents from different types of industries) that connect the river to adjacent Beels, including Mokosh Beel, by entering floodwaters from the upstream portions of the Bangshi River. Surface water samples (N = 63) were collected from 21 preselected locations, as shown in Fig. 1. GPS readings at each sampling point were recorded using GPS. The GPS locations of sampling points are shown in the supplementary section (Table S1). The selection of sampling points in this Beel for determining heavy metals in water involved a more targeted approach, focused on identifying areas most likely affected by contamination and understanding the distribution of pollutants. In addition, the sampling points were selected for the identification of pollution sources, areas of potential contaminant accumulation, and local environmental conditions.

### 2.2 Collection of water sample

Surface water samples were collected from predetermined locations in the Mokosh Beel (Fig. 1) area under the Gazipur administrative district. A total of 63 surface water samples were collected from 21 preselected locations during the winter season of 2022. The selection of 21 sampling locations within the 40 ha area of Mokosh Beel was designed to ensure comprehensive spatial coverage and representativeness of the site's diverse hydrological and environmental conditions. These locations were strategically distributed to capture variations in pollutant levels influenced by industrial effluents, plastic waste, and hydrological connectivity with the Turag River through the Saturia-Solahati canal. The sampling points targeted key areas, including inflow and outflow zones, stagnant water regions, and potential pollutant accumulation hotspots, providing a detailed understanding of heavy metal distribution. This approach balances spatial resolution with logistical feasibility, ensuring robust identification of pollution sources and the overall contamination profile of the Beel.

Subsequently, collecting surface water samples during winter for heavy metal analysis is a strategic decision influenced by several scientific and environmental factors. The major rationale for sampling in winter is the presence of fewer organic materials in surface water. In winter, lower temperatures result in reduced biological activity, such as algae growth and decomposition of organic matter. This can lead to lower levels of natural organic compounds that might bind to heavy metals and influence their availability. Sampling during this period provides a clearer picture of the actual concentrations of dissolved heavy metals in the water. However, the consecutive sampling points were approximately 300 m apart. Samples were collected in pre-cleaned 500-mL plastic bottles. Three bottles of samples were collected from each sampling location. Samples were placed in an icebox after collection and brought to the laboratory. In the laboratory, samples were filtered through .45 μm hydrophilic H-PTFE filters (MN CHROMAFIL Xtra, Germany). For metal analysis, samples were acidified immediately by adding 2.0 mL of ultrapure nitric acid per bottle and shaken well, followed by preservation in the refrigerator at 4 °C.

### 2.3 Analysis of water quality parameters

Physico-chemical parameters of surface water, such as pH, dissolved oxygen (DO), electrical conductivity (EC), total dissolved solids (TDS), and salinity, were measured on-site immediately after sample collection using portable devices following the standard procedure [15]. pH was measured using a pH meter (Model: HI2002-02 with sensor HI1170, Hanna, Romania). Dissolved oxygen was measured using a DO meter (Model: HI2040 with sensor HI764080, Hanna, Romania). Total dissolved solids (TDS) and electrical conductivity (EC) were measured using an EC meter (Model: HI2030, sensor HI763100, Hanna, Romania). Salinity and TDS were measured by the same device using a conversion factor.

### 2.4 Analysis of potential toxic elements (PTEs)

Prior to the analysis for potential toxic elements, all the water samples were digested following the standard method [15]. In this method, 75 ml of water was taken in a beaker using a measuring cylinder, and then 1 ml of 65 % HNO_3_ was added using a micropipette, followed by heating at ∼100 °C to reduce the volume to 25 ml. Finally, the volume was brought up to 50 ml by adding deionized water. The solution was then filtered with .45 μm filter paper, and 10 ml was taken out to the sample bottle for metal analysis. The concentrations of PTEs were determined by an inductively coupled plasma-optical emission spectrophotometer (ICP-OES, 5800 Agilent, USA) in the Water Quality Research Laboratory, Chemistry Division, Atomic Energy Centre, Dhaka. Certified reference material (NIST 1643e, USA) with a known concentration of the analyzed metals was used as control samples to check the measurement precision. The accuracy of the analysis was ensured by the control sample analysis after every ten samples. The reproducibility of the measurement was checked by measuring every sample at least three times.

### 2.5 Quality control

Three bottles of samples were collected from each sampling location. From the three replicates of analysis, we used the average data for interpretation. A common statistical measure was used to assess the relative variability between replicates. A lower CV (typically <10 %) suggests acceptable consistency [16], and our data were in line with the typical guideline for the coefficient of variance (CV). The limit of detection (LOD) is a key parameter in analytical chemistry, as it helps determine the sensitivity of a method or instrument [17] and is based on statistical multiples of the standard deviation of the baseline noise in the data, often expressed in terms of sigma (σ). To ensure the accuracy and precision of the analytical results, the certified reference material NIST 1643e from the National Institute of Scientific and Technology, USA, was employed in triplicate in this study. The percentage of recovery ranged from 91.7 to 109.2, as detailed in Table 1. The mean elemental abundances obtained from triplicate measurements for NIST 1643e showed good agreement with the corresponding certificate values, within the analytical uncertainty limitations.

**Table 1:** Concentrations of potential toxic metals found in certified reference material (NIST 1643e), recoveries, the best wavelength and the limit of detection (LOD) and limit of quantification (LOQ).

Name of Element	Experimental	Certificate value (μg/L)			Recovery %	Accuracy of Element (%)	Wavelength (best) ICP-OES	LOD (3σ)
	Value (μg/L)	Conc. (μg/L)	±	SD				
As (μg/L)	63.9	58.9	±	.70	108	8.43	As 188.98 nm	3.81
Cd (μg/L)	6.80	6.41	±	.07	106	6.12	Cd 228.80 nm	3.83
Co (μg/L)	26.9	26.4	±	.32	101	1.89	Co 238.89 nm	2.43
Cr (μg/L)	21.5	19.9	±	.23	108	8.09	Cr 267.71 nm	3.08
Cu (μg/L)	23.8	22.2	±	.31	107	7.43	Cu 327.39 nm	.94
Mn (μg/L)	35.5	38.0	±	.44	93.4	−6.58	Mn 260.56 nm	2.03
Ni (μg/L)	58.5	60.9	±	.67	96.1	−3.88	Ni 231.60 nm	1.98
Pb (μg/L)	20.8	19.2	±	.20	108	8.56	Pb 283.30 nm	3.80
Sb (μg/L)	56.6	56.9	±	.60	99.4	−.58	Sb 231.14 nm	8.53
Zn (μg/L)	81.5	76.5	±	2.10	106	6.48	Zn 206.20 nm	4.10

### 2.6 Pollution assessment of toxic elements

#### 2.6.1 Heavy metal pollution index

The Heavy Metal Pollution Index (HPI) reflects the cumulative influence of individual heavy metals on the entire water quality and therefore indicates the pollution level concerning these heavy metals [18]. HPI is calculated by combining the following three equations (i, ii, & iii):

(1) $$HPI=\frac{\sum_{i=1}^{n}\;\left(W_{i}Q_{i}\right)}{\sum_{i=1}^{n}W_{i}}$$

(2) $$Q_{i}=\frac{C_{i}}{S_{i}}\times 100$$

(3) $$W_{i}=\frac{K}{S_{i}}$$

here, n represents the total number of analyzed heavy metals, W_i_ represents the unit weight of the ith component of metals, Q_i_ corresponds to the sub-index, C_i_ represents the monitoring concentrations of the individual heavy metals, and S_i_ corresponds to the drinking water standard. k is a proportionality constant set to 1. Following the calculations with k set to 1, the resulting HPI values are then categorized into four levels of metal pollution: i) HPI values less than 15 indicate a very low level of pollution; ii) HPI values ranging from 15 to 30 indicate low to moderate pollution; iii) HPI values ranging from 30 to 100 indicate moderate to heavy pollution; and iv) HPI values higher than 100 indicate a very high degree of pollution, which is generally considered the critical limit for HPI [18].

#### 2.6.2 Heavy metal evaluation index (HEI)

The heavy metal evaluation index (HEI) is a numerical tool used to assess the overall pollution level of water based on the concentration of various heavy metals. It provides a cumulative measure by assigning a weight to each metal according to its potential environmental and health risks. The HEI helps to categorize water quality into different risk levels, allowing for a clear evaluation of contamination [19]. This index is widely used in environmental monitoring to compare pollution levels across sites and over time, offering valuable insights for water quality management and public health protection. The heavy metal evaluation index (HEI) determines the metal pollution level based on the suspended heavy metals in water. The heavy metal evaluation index (HEI) was used for evaluating the overall water quality as demonstrated by Edet and Offiong [20]:

The following equation (iv) was used to calculate the HEI index:

(4) $$\text{HEI}=\sum_{i=1}^{n}\frac{C_{i}}{MAC_{i}}$$

The following equation (iv) was used to calculate the HEI index, where C_i_ represents the monitored concentration and MAC_i_ represents the maximum permissible concentration level of heavy metals. The classification of surface water quality according to HEI results in three categories: i) HEI values less than 10 indicate low pollution; ii) HEI values ranging from 10 to 20 indicate moderate pollution; and iii) HEI values greater than 20 indicate a high degree of pollution [18].

#### 2.6.3 Single-factor pollution index (P_i_)

The single-factor pollution index (P_i_) explores the hazard level of a single particular pollutant to the overall water quality and is calculated by dividing the analyzed concentration with the standard value of that particular pollutant [21]. This can be expressed by equation (v):

(5) $$P_{i}=\frac{C_{i}}{S_{i}}$$

where C_i_ represents the actual monitored concentration and S_i_ represents the standard concentration of the heavy metals. The surface water pollution level based on the single-factor pollution index is categorized into: i) pure, when Pi is less than 1; ii) low, when P_i_ ranges between 1 and 2; iii) moderate, when P_i_ ranges between 2 and 3; and iv) high, when P_i_ is greater than 3 [22].

#### 2.6.4 Nemerow pollution index (N_P_)

The Nemerow Pollution Index (N_P_) provides a comprehensive pollution evaluation of the overall water quality, derived from various pollutants, by combining both the average and maximum values obtained from the single-factor pollution index [18]. Therefore, NP is calculated by using equation (vi):

(6) $$N_{P}=\sqrt{\frac{{\left(P_{i}\right)}_{m}^{2}+{\left(P_{i}\right)}_{a}^{2}}{2}}$$

where (P_i_)_m_ and (P_i_)_a_ represent the maximum and average values of the single-factor pollution index, respectively. N_P_ is used to categorize surface water pollution levels into five grades [18]: i) clean when N_P_ is less than .7; ii) clean but when N_P_ ranges between .7 and 1; iii) mild pollution when N_P_ ranges between 1 and 2; iv) moderate pollution when N_P_ ranges between 2 and 3; and v) heavy pollution when N_P_ is greater than 3.

### 2.7 Source identification

Principal component analysis (PCA), cluster analysis (CA), and Pearson bivariate correlation matrix (CM) were performed to obtain informative analytical data sets and procure insight into the metal distribution patterns using SPSS V24. Hierarchical cluster analysis was applied using Ward's method, and the results were expressed in a dendrogram to represent a system of organized variables in which each group exhibits common characteristics. The interrelationship among the analyzed metals in the samples was evaluated using a Pearson bivariate correlation matrix (CM) to identify the likely sources of sample contamination [23]. A strong correlation indicated a similar source, whereas no correlation indicated a single source.

### 2.8 Ecological risk assessment

Excessive toxic metal contents in the surface water could affect the ecological health of the aquatic organisms. Therefore, the potential ecological risk index (PERI) was used in this study as a distinctive tool to quantitatively assess the potential ecological health risk of the aquatic creatures imposed by potential toxic elements in the Mokosh Beel water [24]. This model calculates the Ecological Risk Index (ER_i_), incorporating the measured concentrations of heavy metals, their toxic response factors, and a pre-determined baseline level [25,26]. By summing the risk contributions of individual metals, the ERI provides an overall assessment of ecological risk. This method enables the identification of metals posing the highest threat to aquatic organisms and helps prioritize mitigation efforts [19]. The model is widely recognized for its ability to offer a standardized and quantitative approach to evaluating ecological risks, ensuring relevance and comparability across similar studies [[27], [28], [29]]. According to Hakanson [30], the potential ecological risk factor (ER_i_) of each metal can be evaluated by using the following equation (vii):

(7) $$ER_{i}=T_{r}\times \frac{C_{i}}{\;C_{f}}$$

where T_r_ stands for the toxic response factor of each element. The T_r_ values for the given elements Cu, Pb, Zn, Ni, Cd, Cr, and As are 2, 5, 5, 1, 30, 10, and 5, respectively [31]; those for Mn and Co are 1 and 5, respectively; C_i_ is the monitored concentration of each element in the water, and C_f_ is the standard pre-industrial reference concentration of each element (in mg kg⁻^1^): 15 for As, 25 for Co, 1 for Cd, 90 for Cr, 175 for Zn, 70 for Pb, 50 for Cu, 68 for Ni, and 850 for Mn [24]. The potential ecological risk index (PERI) is then calculated by summing all the potential ecological risk factors of each heavy metal as follows (equation viii), and the risk levels based on the PERI can be categorized (Table 2) into four major grades [24,31].

(8) $$\text{PERI}=\sum_{i=1}^{n}ER_{i}$$

**Table 2:** The risk levels based on the PERI can be categorized into four major grades (Niu et al., 2019 [24]).

PERI value	Remarks
PERI <150	Low risk
150 ≤ PERI <300	Moderate risk
300 ≤ PERI <600	High risk
PERI ≥600	Extremely high risk

### 2.9 Health risk assessment

Health risk assessment evaluates the extent of health impacts on the human body caused by exposure to various toxic trace metals. This method is primarily based on the assessment of carcinogenic and non-carcinogenic risk levels, which in turn are determined by considering direct oral ingestion and dermal contact as the primary routes of exposure to the trace elements [32]. The health impacts due to exposure to trace elements can be different for different age groups, and hence the health risk has been assessed in this study for both adults and children individually.

#### 2.9.1 Non-carcinogenic risk assessment

The assessment of non-carcinogenic risk involved the calculation of the Hazard Quotient (HQ) and Hazard Index (HI). HQ was computed for both adults and children considering the direct oral ingestion and dermal pathways. This calculation was performed by comparing the average daily dose (ADD) to the reference dose (RfD) using the following ix, x, and xi equation [33]:

(9) $${\text{HQ}}_{\text{ingestion}}=\frac{ADD_{ingestion}}{RfD_{ingestion}}$$

(10) $${\text{HQ}}_{\text{dermal}}=\frac{ADD_{dermal}}{RfD_{dermal}}$$

(11) $$\text{HQ}={\text{HQ}}_{\text{ingestion}}+{\text{HQ}}_{\text{dermal}}$$

The average daily dose for oral ingestion and dermal contact is expressed for both adults and children using the following two (xii & xiii) formulas [33].

(12) $${\text{ADD}}_{\text{ingestion}}=\frac{C_{w}\times \text{IR}\times \text{EF}\times \text{ED}\times \text{Ab}s_{g}\;}{\text{BW}\times \text{AT}}$$

(13) $${\text{ADD}}_{\text{dermal}}=\frac{C_{w}\times SA\times K_{p}\times EF\times ET\times ED\times 10^{-3}\;}{BW\times AT}$$

In this study, several parameters were considered for calculating the average daily dose (ADD) for ingestion and dermal absorption. These parameters include C_w_, which stands for the average concentration of estimated metals in water (μg/L); IR, representing the ingestion rate (.64 L/day and 2 L/day for children and adults respectively); EF, exposure frequency (350 days/year); ED, exposure duration (6 years and 70 years for children and adults respectively); Abs_g_, the gastrointestinal absorption factor; BW, average body weight (70 kg and 15 kg for adult and children respectively); SA, exposed skin area (18,000 cm^2^ for adults and 6600 cm^2^ for children); K_p_, the dermal permeability coefficient in water (cm/h); AT, averaging time (365 days/year × 70 years and 365 days/year × 6 years for adults and children respectively); and ET, exposure time (.58 h/day for adults and 1 h/day for children). These parameters were essential for assessing non-carcinogenic risk through HQ and HI calculations [33].

The Hazard index (HI) indicates the overall potential non-carcinogenic health risks, which is calculated by summing up the hazard quotients for ingestion and dermal pathways (equation xiv). Potential non-carcinogenic health risk is only indicated when HQ and HI values exceed 1 [33].

(14) $$\text{HI}=\sum_{i=1}^{n}\left({\text{HQ}}_{\text{ingestion}}+{\text{HQ}}_{\text{dermal}}\right)\;$$

#### 2.9.2 Carcinogenic risk assessment

Carcinogenic risk refers to the likelihood of developing cancer in the human body due to exposure to cancer-causing toxic metals [34]. Carcinogenic risk is also determined for both the dermal and oral ingestion routes using the following two (xv & xvi) formulas:

(15) $${\text{CR}}_{\text{ingestion}}={\text{ADD}}_{\text{ingestion}}\times {\text{CSF}}_{\text{ingestion}}$$

(16) $${\text{CR}}_{\text{dermal}}={\text{ADD}}_{\text{dermal}}\times {\text{CSF}}_{\text{dermal}}$$

Here, CSF_ingestion_ and CSF_dermal_ represent the cancer slope factor for direct oral and dermal pathways, respectively. In our study, the CR_ingestion_ was evaluated for Ni, Cd, As, Pb, and Cr, in which their corresponding cancer slope factors are 9.1 × 10^−4^, .041, 1.5 × 10^−3^, 8.5 × 10^−3^, and 3.8 × 10^−4^ (mg/kg/day)^−1^, respectively. CSF_dermal_ other than As was not available, and hence CR_dermal_ was only evaluated for As. The cancer slope factor of As for dermal pathways is 3.6 × 10^−3^ (mg/kg/day)^−1^ [33].

The total potential carcinogenic risk can be calculated as follows (eq. xvii):

(17) $$\text{CR}={\text{CR}}_{\text{ingestion}}+{\text{CR}}_{\text{dermal}}$$

According to Ref. [35], the potential carcinogenic risk is tolerable when it is less than 1.0 × 10^−4^.

## 3 Result and discussion

### 3.1 Water quality parameters

The physicochemical parameters such as pH, DO, EC, TDS, and salinity at various sampling points of Mokosh Beel are shown in Table S2. The pH values in various sampling locations varied between 7.19 (S-4) and 7.85 (S-13), and all these pH measurements fall within the standard permissible limit suggested by WHO [36] and ECR [37]. The pH values above 7 indicate a slight alkalinity of all the water samples. This alkalinity may be due to the presence of aluminum hydroxide originating from the effluent of an aluminum factory near Mokosh Beel [38]. The pH of water bodies may also be influenced by various chemical and biochemical reactions. The tolerable limit of pH ranges from 5 to 9 for most fish, and a smaller change in pH does not impose any harm to aquatic life [22]. The average dissolved oxygen (DO) at different sampling points was found to be 3.69 mg/L, with a range from 1.43 mg/L to 6.19 mg/L. The DO values of all the sampling locations except S-1 fall below the WHO [36] guideline value of 6 mg/L. These low values of DO may be due to effluent from tannery industries, dyeing, and printing industries near Mokosh Beel containing biodegradable organic compounds. When these effluents are discharged into water bodies, microorganisms consume oxygen, which reduces the dissolved oxygen levels [23]. However, organic dyes may accumulate in water and consequently lower the oxygen concentration by inhibiting plant photosynthesis. The electrical conductivity (EC) was found to vary from 2050 μS/cm to 3410 μS/cm, where the EC values in all sampling locations exceeded the maximum allowable limit of 1500 μS/cm set by WHO [36] and USEPA [35]. In most cases, the values exceeded the guidelines by nearly three times. The degree of water pollution is directly related to electrical conductivity, since electrical conductivity is associated with the number of dissolved metal constituents in water [22]. Industrial effluent may contain metals (lead, copper, cadmium, etc.) and salts (chlorides, sulphates, nitrates, and carbonates) that contribute to an increase in electrical conductivity when present in the water as dissolved ions [39]. The average total dissolved solids (TDS) value in this study was found to be 1548 mg/L, and all the TDS values from various sampling locations exceeded the WHO [36] guideline value of 1000 mg/L. Excessive TDS levels in water may disrupt osmoregulation and physiological functions of fish and other aquatic organisms [39]. High TDS concentrations may also affect human health. The present study showed a variation of salinity from 990 mg/L to 1750 mg/L. In most of the sampling points, these values exceeded the standard limit as suggested by WHO [36]. Sampling point S-11 was recorded to have the highest salinity, whereas the lowest salinity was found at S-3. High salinity in water bodies can be harmful to aquatic ecosystems.

### 3.2 Distribution of potentially toxic elements

**Fig. 2:**
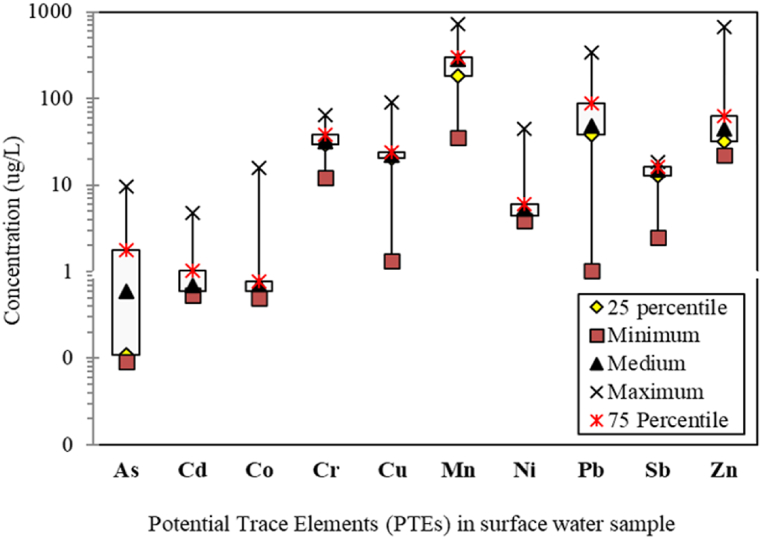
Box-whisker plot representing the distribution of potentially toxic elements in the surface water of Mokosh Beel, Gazipur, Bangladesh.

**Fig. 3:**
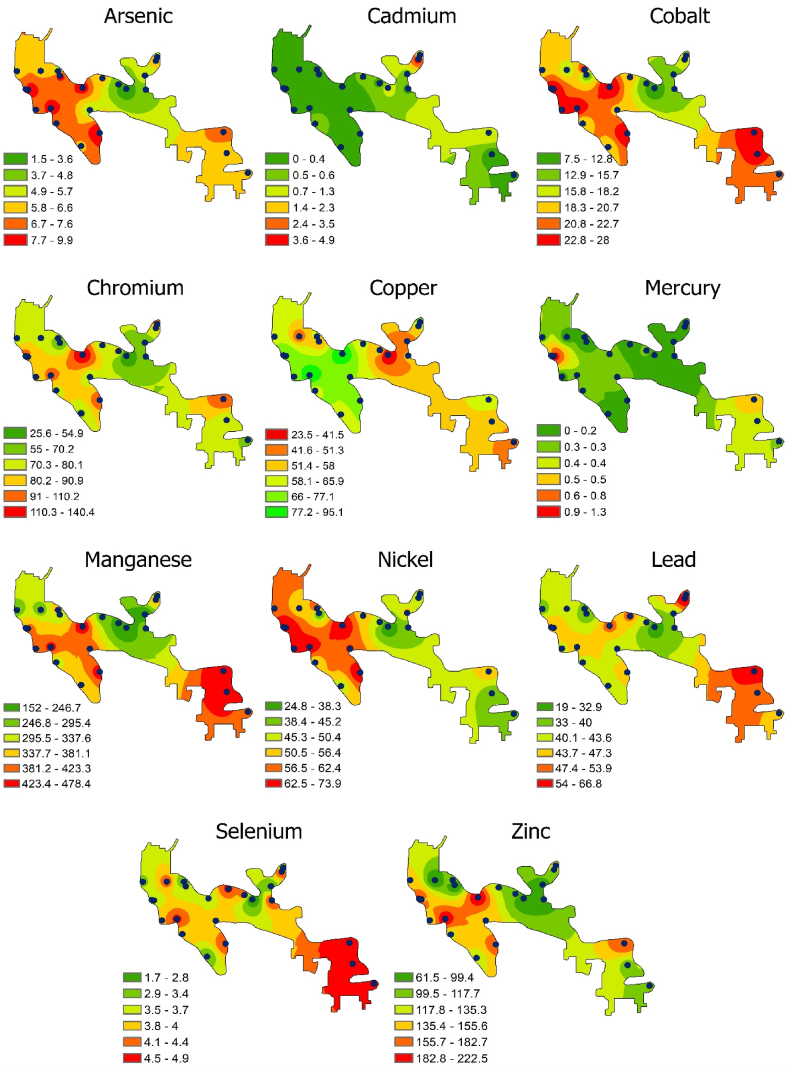
Spatial distribution of PTEs in the surface water of Mokosh Beel, Gazipur, Bangladesh.

The PTEs (Mn, Cd, Co, As, Cr, Cu, Zn, Ni, Sb, and Pb) concentrations and their distributions in Mokosh Beel water are presented in Table 3. Table 3 illustrates the comparison of PTE concentrations in Mokosh Beel water with other surface water sources from local and international literature along with their national and international guideline values. , the mean heavy metal concentrations in the investigated surface water are also shown in the box and whisker plot (Fig. 2), which range from .09 to 9.73, .53 to 4.81, .50 to 15.93, 12.24 to 64.7, 1.32 to 91.01, 35.04 to 724.3, 3.82 to 45.01, 1.02 to 341.29, 2.51 to 18.5, and 21.92–671.79 μg/L for the concentrations of As, Cd, Co, Cr, Cu, Mn, Ni, Pb, Sb, and Zn, respectively. In the diagram shown in Fig. 2, the triangular symbol within the box denotes the median value, while the vertical bars represent the range of data spanning from the 25th to the 75th percentile. The study showed that Mn had the highest concentration in the Mokosh Beel samples, followed in descending order by Zn, Pb, Cr, Cu, Sb, Ni, Co, As, and Cd, Beel respectively. However, the spatial distribution of PTEs around the various locations of Mokosh Beel water is depicted in Fig. 3.

**Table 3:** Comparison of the potential toxic element's concentration (μg/L) in surface water of Mokosh Beel with guideline value and other surface water from national and international literature.

Recommended Limits and Literature Data	As	Mn	Pb	Ni	Cu	Cd	Sb	Cr	Co	Zn
Mokosh Beel, Gazipur, Bangladesh (Present study)	1.43	269.1	71.47	9.08	25.5	1.04	14.1	33.25	1.79	87.61
**Recommended Limits**										
WHO [36]	10	400	10	70	2000	3	20	50	100	3000
ECR [37]	50	100	50	100	1000	5	–	50	–	5000
EPA [40]	10	50	10	20	2000	5	10	50	–	5000
USEPA [35]	10	–	15	–	1300	5	6	10	100	–
**Literature Data**										
Baro haor, Kishoreganj, Bangladesh [41]	–	156.1	4.05	–	17.5	1.8	–	–	–	4.2
Shitalakhya River, Bangladesh [42]	–	–	13.16	–	24.6	.64	–	38.3	–	75.4
Chalan Beel, Bangladesh (Salam et al., 2021)	–	8.6	51.42	60.4	4.7	6.2	–	–	–	10.7
Brahmaputra River, Bangladesh [43]	–	1440	110	440	120	1	–	10	200	10
Karnofuly River, Bangladesh (Islam et al., 2013)	–	120	140	–	50	10	–	250	–	280
Buriganga River, Bangladesh [44]	134	157	112	150	–	59	–	114	–	332
Turag River, Bangladesh (Hafizur et al., 2017)	–	–	3.82	–	33.5	.51	–	–	–	–
Dhaleshwari River, Bangladesh [45]	–	–	50	7	150	6	–	440	–	–
Meghna River, Bangladesh [24]	24	–	9	–	–	18	–	45	–	–
Meghna River, Bangladesh [46]	–	9	BDL	BDL	–	3	–	35	–	36
Ganga River, India [47]	–	72	5	–	–	–	–	33	–	289
Gomti River, India (Gupta et al., 2015)	–	30	20	–	–	–	–	100	–	70
Yangtze River, China (Wu et al., 2009)	13.2	5.4	55.1	13.4	10.7	4.7	65.3	20.9	–	9.4
Coruh river basin, Turkey (Bilgin et al., 2016)	–	91	14	–	–	–	–	.7	–	92
Commodore Channel, Nigeria [48]	–		1232	–	–	53	–	–	75	193
Major River, Argentina (Avigliano and Schenone, 2015)	–	22	1	–	–	–	–	–	–	22
Pardo River, Brazil [49]	–	35	3	–	–	–	–	.8	–	12
Xiangjiang river, China [50]	–	–	2	–	–	–	–	6	–	84

#### 3.2.1 Lead (Pb)

This study recorded the average concentration of lead (Pb) as 71.47 μg/L, which exceeded all the national and international standard limits such as ECR [37], WHO [36], EPA [40], and USEPA [35]. The finding was also comparatively higher than the tabulated literature data (Table 3) except for the Brahmaputra River, Bangladesh [43], the Buriganga River, Bangladesh [44], and the Commodore Channel, Nigeria [48]. The spatial distribution map shows that a high concentration of lead (Pb) is scattered in the central region of the study area. The highest Pb concentration was recorded at sampling point S-19 (341.29 μg/L), which might be attributed to the discharge of effluents from the tannery, dyeing, and printing industries located near this region [22,23,51]. Excessive concentrations of Pb affect the central nervous system, especially in young children. Moreover, chronic exposure to lead (Pb) may impose a number of health issues, including cancer and anemia in adults, while young children may face hormonal imbalances, reproductive harm, and a reduction in IQ [22].

#### 3.2.2 Manganese (Mn)

The average manganese (Mn) concentration was found in this study to be 269.13 μg/L, which not only exceeded the ECR [37], EPA [40], and USEPA [35] limits but also the tabulated national and international literature data except for Brahmaputra River, Bangladesh [43] and WHO [36] guidelines. The elevated concentrations of Mn were primarily located in the northern, eastern, and northwestern regions of the study area (Fig. 3). The highest concentration of manganese (Mn) was observed at sampling points S-19 (724.30 μg/L) and S-3 (571.03 μg/L). These two hotspots were detected in the northern and eastern regions, respectively. The aluminum industries near Mokosh Beel may be responsible for the elevated content of Mn, since the effluents from aluminum industries reduce pH and consequently release manganese into the water [38]. Although Mn is an essential nutrient, long-term exposure to Mn can affect nerve, liver, and kidney function [22].

#### 3.2.3 Cadmium (Cd)

This study recorded a variation of cadmium concentration from .531 to 4.805 μg/L (Fig. 2), with all the recorded concentrations at each sampling point except S-19 were being below the limits suggested by ECR [37], WHO [36], EPA [40], and USEPA [35]. However, the mean concentration of Cd was found to be 1.04 μg/L, which is considered to be very low in comparison to other literature data (Table 3). Therefore, it appears that the Mokosh Beel water is almost free from pollution by cadmium (Cd)-containing compounds. High contents of Cd were enriched at the northern edge of Mokosh Beel, which might originate from the weathering of soils and minerals, atmospheric deposition from the mining of non-ferrous metals, and effluent from the steel and iron industries [52]. Cd is regarded as a non-essential element and is very toxic to aquatic organisms and plants. Cd exerts its toxic effect by interfering with metabolic processes in plants and bioaccumulating in aquatic organisms [51]. Cadmium (Cd) is indeed highly toxic to humans, and its detrimental effects can include conditions such as renal damage, “itai-itai” disease, hypertension, emphysema, and testicular atrophy.

#### 3.2.4 Antimony (Sb)

This study recorded the mean concentration of antimony (Sb) as 14.119 μg/L (2.514–18.498 μg/L), which exceeded the EPA [40] and USEPA [35] limits but was within the limit of the guideline value suggested by WHO [36]. The high contents of Sb were scattered within the western to central zone of the study area. The sampling point S-11 was recorded to have the highest concentration of Sb, which is located in the western part of the study area (Fig. 3). Additional industrial activities related to antimony (Sb) contamination might be present in the western part. However, antimony (Sb) contamination in surface water can originate from various industrial processes, especially the textile and manufacturing sectors. In textile production, Sb is commonly used as a catalyst in polyester manufacturing, and its release occurs through wastewater discharges during dyeing, finishing, and washing processes [53]. The electronics, flame retardant, and battery industries also contribute to Sb contamination through industrial effluents and improper waste disposal. Sb is considered a genotoxic element, and elevated levels of this element in the aquatic ecosystem can pose a significant threat to human health [54].

#### 3.2.5 Chromium (Cr)

Chromium (Cr) does not serve any essential functions in the human body but can have harmful impacts on body tissues. According to this study, the mean concentration of chromium (Cr) was observed to be 33.203 μg/L (Table 3), which is three times higher than the USEPA [35] recommended value but falls below the values recommended by WHO [36], ECR [37], and EPA [40]. The highest concentration of Cr was observed at the sampling point S-19 (64.701 μg/L), which lies at the northern edge of Mokosh Beel. Interestingly, the mean concentration of Cr recorded in this study exceeded most of the international literature data but fell below all the national literature data (Table 3) except for the Brahmaputra River, Bangladesh [43]. Chromium could originate mainly from the tannery industry near Mokosh Beel. Cr is a very toxic element, and due to its carcinogenic effect, chromium (Cr) should be removed from water before consumption to ensure safe surface water quality [22,51].

#### 3.2.6 Copper (Cu)

In the present study, the concentration of Cu ranged between 1.317 and 91.009 μg/L (Fig. 2), which all the recorded concentrations were far below the standard limits set by ECR [37], WHO [36], EPA [40], and USEPA [35]. The spatial distribution map implies that the respective concentrations of Cu coincided with the central zone of Mokosh Beel, which might originate from leather tanning, dyes, and textile industries located in this region [33]. Although Cu is not significantly harmful at low doses, it imposes a number of health issues at high doses, such as kidney failure, organ impairment, vomiting, nausea, haemolytic jaundice, and central nervous system disorder [22].

#### 3.2.7 Nickel (Ni)

This study revealed the concentration of Ni varied between 3.822 and 45.014 μg/L (Fig. 2), with all the recorded concentrations at each sampling point except S-7 and S-19 were being below the limits suggested by ECR [37], WHO [36], and EPA [40]. However, the mean concentration of Ni recorded in this study was found to be several times lower compared with the tabulated existing literature data (Table 3). Apart from this, the high contents of Ni were scattered from the northern to the central zone of the study area. Ni may be discharged into water bodies as industrial effluent from certain industries such as chemicals, automobile batteries, steel alloys, and surgical instruments [38]. Long-term exposure to Ni causes chronic non-cancer health impacts. Ni also has a carcinogenic effect, and inhalation of Ni can cause cancer of the lungs, nose, sinuses, throat, and stomach.

#### 3.2.8 Arsenic (As)

Arsenic ingestion induces perilous chronic arsenicism in the human body and consequently results in a number of serious health issues such as skin and lung cancers, bladder cancer, dermal lesions, peripheral neuropathy, and peripheral vascular diseases. Therefore, it is crucial to minimize exposure to this highly toxic element. The average concentration found in this study was 1.418 μg/L (.086–9.726 μg/L) (Table 3), which was less than all the guideline values suggested by WHO [36], ECR [37], EPA [40], and USEPA [35]. The low levels of As in this study might be due to the presence of the least amount of As in the geological formations and the high water flow in Mokosh Beel [55]. Only the northern edge of the study area was slightly contaminated by arsenic, which might be due to the geological conditions of the particular area. The limits of As in natural waters are generally below 2 μg/L, so the water in Mokosh Beel is relatively safe from As contamination.

#### 3.2.9 Cobalt (Co)

The average concentration of Co found in Mokosh Beel was 1.792 μg/L, which was about 50 times lower than the standard values suggested by WHO [36] and USEPA [35], indicating no pollution imposed by this trace metal. The sublime concentration of Co coincided with the northern edge of Mokosh Beel, which might have originated from industrial effluent, urban runoff, and geological conditions in this particular area [33]. Excessive cobalt (Co) intake can be pernicious as it is a carcinogen, and hence the therapeutic use of this toxicant should be under medical supervision.

#### 3.2.10 Zinc (Zn)

Zn is essential for physiological activities, but their excess bioaccumulation in the human body causes zinc toxicity. This toxic element may originate from galvanic corrosion, domestic sewage, and different forms of organic materials. In the current study, the concentration of zinc (Zn) was found to vary within the range of 21.92 μg/L to 671.79 μg/L (Fig. 2), which was far below the guideline value as recommended by WHO [36], ECR [37], and EPA [40]. The relative abundances of Zn were also scattered from the northern to the central parts of the study area. The sublime concentration of Zn was found in this study at sampling point S-19, whereas the lowest concentration of Zn was recorded at S-20. The low levels of Zn demonstrate that Mokosh Beel water is comparatively free from Zn toxicity.

### 3.3 Assessment of pollution indices

The water pollution indices (HPI, HEI, Pi, NP) were used in this study to assess the pollution levels in Mokosh Beel, providing a comprehensive evaluation of the overall water quality. It should be remembered that no studies have been done yet to assess these pollution indices in Mokosh Beel water. This will be the first study to assess these pollution indices that were calculated for several toxic metals such as Cr, Cd, Co, Cu, As, Ni, Pb, Zn, Mn, and Sb using the national standard guideline values [37] and international standard guideline values [36].

**Fig. 4:**
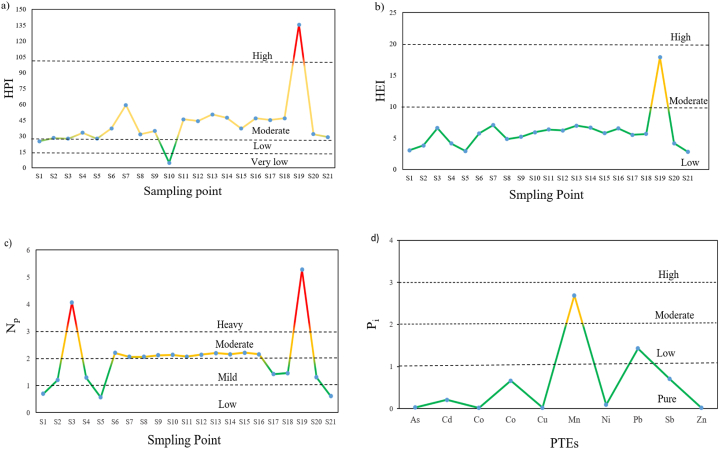
(a) Heavy metal pollution Index, **(b)** heavy metal evaluation index, **(c)** Nemerow pollution index at various sampling points, and **(d)** mean single factor pollution index of PTEs.

The Heavy Metal Pollution Index (HPI) values were computed for all 21 water samples, and the results are presented in Fig. 4a. The average HPI value observed in Mokosh Beel water was 43.140, as displayed in Table S3. Importantly, this value is lower than the critical index threshold of 100, suggesting that the water quality in Mokosh Beel, in terms of heavy metal pollution, is within an acceptable range [22]. The HPI values revealed that almost all the sampling points (95 %) fell below the critical index value; only sampling point S-19 was the exception. The HPI values at the sampling points S-1, S-2, S-3, S-5, S-10, and S-21 were below 30, indicating that these sampling areas have a low degree of metal pollution. Sampling point S-10 was found to be the lowest (4.65), and therefore, the water in these areas can be considered relatively pure for consumption. On the other hand, HPI values at the sampling points S-7, S-13, and S-19 were above 50, indicating relatively higher levels of heavy metal pollution in these areas. Sampling point S-19 was found to have the highest HPI value (135.52), which is considered to be the most polluted area. The rest of the sampling points were moderately polluted with respect to heavy metals. Therefore, the surface water from all the sampling sites except S-19 can be used for domestic and other purposes with respect to heavy metals. A similar kind of study was carried out by Bhuiyaan et al. (2015) to compute the level of pollution in the Buriganga River, where the HPI values varied from −.12 to 455.77 with a mean value of 250.77. According to these results, about 87 % of the samples were above the critical limit. Recently, Haque et al. [56] executed a similar study in the Ganga River, where HPI values ranged from 1.92 to 26.47, and 75 % of the samples fell into the low level of pollution. Moniruzzaman et al. (2020) studied the water quality of different aquifers in the vicinity of our study area and reported that the HPI values for all the shallow and midway groundwater exceed 100. This was due to elevated levels of toxic metals and anthropogenic pollutants and is therefore unsuited for drinking purposes. On the other hand, the deep wells and river waters have low levels of HPI, making them suitable for drinking and other purposes. Yüksel et al. (2020) reported the water quality of Çavuşlu Stream at Giresun in Türkiye, where all the sampling stations along with tap water extracted from the stream had HPI <100 and exhibited a low level of pollution. Further, Karadeniz et al. [57] calculated HPI as 45 to reveal the water quality and toxicological risks in plateaus of Giresun Province.

The heavy metal evaluation index (HEI) was determined in this study to explore the overall quality of Mokosh Beel water by assessing the pollution level resulting from the loading of heavy metal contents. The mean HEI value of the collected sample was calculated to be 5.88 (Table S3), which is under the category of a very low degree of pollution (<10). Fig. 4b shows the HEI values of all the sampling points (95 %) except S-19 were below 10, indicating that the majority of the sampling area was not affected by heavy metals, and water quality in these particular regions can be considered safe to humans and overall ecosystems. The lowest HEI value, 2.768, was calculated at sampling point S-21, while the highest value, 17.858, was calculated at sampling point S-19. Bhuiyaan et al. (2015) investigated HEI in the Buriganga River, in which these values varied from 5.34 to 43.49, and about 66 % of the samples exceeded the critical limit of 20. According to Haque et al. [56], HEI values in the Ganga River ranged from .52 to 3.35 and disclosed the majority of the sampling sites were free from metal pollution. Moniruzzaman et al. (2020) reported a high level of HEI in the middle and shallow aquifer in Tangail, whereas deep wells have a considerably low level of HEI. According to Tokatli [58], HEI values of all the sampling areas from the Ergene River, Meriç River, and Dam Lakes were below 10, indicating a very low degree of pollution in all the sampling stations. Moreover, Yüksel et al. (2020) reported a very low level of contamination based on HEI at 3 out of 4 stations in the Çavuşlu Stream of Türkiye.

The Nemerow pollution index (NP) was used to observe how multi-components contaminate water at each sampling site [56]. The variation of the Nemerow pollution index at various sampling points is presented in Fig. 4c. The mean value of NP was found to be 1.97 (Table S3), indicating that the water in Mokosh Beel was mild to moderately polluted with respect to heavy metals. NP values revealed that about 14 % of the total samples (S-1, S-5, S-21) fell into the category of clean water, about 24 % of samples (S-2, S-4, S-17, S-18, S-20) fell into the category of mild pollution, about 52 % of samples were categorized as moderately polluted water, and only 10 % of the total samples (S-3, S-19) were categorized as highly polluted water. Islam et al. [59] explored the Nemerow pollution index on the Bay of Bengal coast of Bangladesh, where Np values were 17.8 for Cox's Bazar coast, 23.8 for Chittagong coast, 7.8 for Meghna Estuary, and 11.6 for Sundarban's coast. According to these results, Cox's Bazar and Chittagong coast were highly polluted, while Meghna Estuary and Sundarban coast were moderately polluted by heavy metals. Haque et al. [56] reported Np values in the Ganga River ranged from .09 to .67, indicating no metal pollution in the river water.

The mean single-factor pollution index (Pi) for each heavy metal is displayed in Fig. 4d. The figure shows Mn was ranked highest among the heavy metals for the contribution of pollution in Mokosh Beel water. The mean values of Pi for manganese (Mn) and lead (Pb) were found to exceed 1, whereas the mean Pi for other metals was found to be less than 1, signifying that Mn and Pb were the major components responsible for polluting the water in Mokosh Beel. However, the variation of the single-factor pollution index at each sampling point is displayed in Table S4. The Pi for Mn was greater than 1 in almost all the sampling points except S-1, S-5, and S-21. The values revealed that about 14 % of the total samples were not affected, 24 % of samples were slightly affected, 43 % of samples were moderately affected, and about 19 % of samples were highly affected by Mn. The Pi for Pb showed that about 48 % of samples were not affected, 43 % of samples were slightly affected, 10 % of samples were moderately affected, and only sample S-19 was highly affected by Pb. The Pi for Cr showed that almost all the sampling points except S-19 were not affected by Cr. However, the Pi for the rest of the heavy metals was less than 1 for all the recorded sampling points. Thus, these heavy metals were not responsible for the pollution of Mokosh Beel water. Haque et al. [56] studied the quality of the Ganga River concerning heavy metals and reported that the single-factor pollution index for all the metals was less than unity and indicated no pollution occurred by the analyzed heavy metals. Moniruzzaman et al. [60] investigated the water quality of different aquifers near our study region and disclosed that Fe and As were primarily culpable for metal pollution. The single-factor pollution index in the Halda River revealed that the river water was highly contaminated with respect to Cd, As, Cu, and Pb but relatively pure with respect to Cr and Zn [22].

### 3.4 Source identification

#### 3.4.1 Principal components analysis (PCA)

**Fig. 5:**
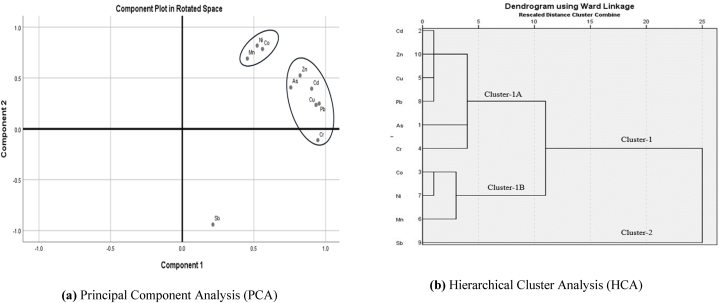
Source identification of potential toxic elements (PTEs) in surface water sample using (a) Principal Component Analysis (PCA) and **(b)** Hierarchical Cluster Analysis (HCA).

PCA is a well-accepted method for source apportionment of metals in pit-surface-groundwater [61]. The accumulation of metals in groundwater primarily comes from both natural and human activities. Varimax with the Kaiser normalized rotation method was used for principal component analysis of metals in the study area. The KMO of .712 signifies the PCA analysis. Table S5 shows the rotated component matrix. The eigenvalues greater than 1 were considered for further analysis and evaluation of PCA [62]. The loading factor values greater than .750 were considered significant for the interpretation of source apportionment [61]. The total variances were explained by two principal components or factors (Fig. S1). A total of 89.56 % of variances were explained by two factors; PC1 explained 71.46 % and PC2 explained 18.11 % of the total variances. The As, Cd, Cr, Cu, Pb, and Zn were clustered in PC1, and Co, Ni, and Sb were clustered in PC2. However, the rotated plot (Fig. 5a) showed that Ni, Co, and Mn were concentrated in one place and As, Zn, Cd, Cu, and Pb were clustered in one place. The Cr and Sb were placed solely. The distribution on the plot suggested that Mn, Ni, and Co are derived from one source, whereas As, Zn, Cd, Cu, and Pb are derived from another source. Finally, Cr and Sb appear to be derived from other sources.

#### 3.4.2 Hierarchical cluster analysis (HCA)

Hierarchical cluster analysis (HCA) revealed two major groups, similar to the PCA components. One group consists solely of Sb, while the other includes the remaining metals. This second group is further divided into two sub-groups, 1A and 1B. The 1A group comprises Cd, Zn, Cu, Pb, As, and Cr, while the 1B group contains Co, Ni, and Mn. The dendrogram of the cluster analysis (CA) is provided in Fig. 5b.

PCA1 accounts for 71 % of the total variance and is linked to mixed sources. As and Cr likely originated from agrochemical and leather industries [63], while Cd, Zn, Cu, and Pb are probably from industrial sources [64]. PCA2 explains 18 % of the variance, showing a close relation among Co, Ni, and Mn with strong positive weights and Sb with a strong negative weight. Co, Ni, and Mn may result from combined natural and anthropogenic sources, such as agriculture and industries [65]. Toxic elements may have originated from municipal wastes and industries like paint, leather, and pesticides [66]. Saha and Rahman [67] proposed three principal sources of surface water metal contamination: a) industrial effluents (Pb, Ni, Cu, Zn, and As), b) municipal wastes (Cr, Cu, and Mn), and c) atmospheric deposition (Cd). The present study suggests that As and Cr may be released from paint and leather industries, while Cd, Zn, Cu, and Pb may have originated from industrial and atmospheric deposition, and Co, Ni, and Mn may have dissolved from agricultural and industrial sources.

#### 3.4.3 Pearson's correlation analysis

The Pearson correlation coefficient was used to establish the association between the potential toxic metals that were examined in the present investigation (Table 4). The As concentrations showed a significant positive correlation with Cd and Pb. The Cd concentrations had a significant correlation with Cu, Pb, and Zn. Strong, significant positive correlations were observed among Mn, Ni, and Zn with Co. This could be due to the fact that arsenic (As), cadmium (Cd), and lead (Pb) in surface water near industrial areas correlated due to their common industrial sources and similar chemical behaviors [68]. These heavy metals are frequently released together through industrial effluents, including from metal processing, battery manufacturing, and textile industries [69]. Their similar solubility, adsorption, and mobility patterns in aquatic environments contribute to their co-occurrence, often reflecting joint contamination pathways [70].

**Table 4:** Pearsons correlation between the concentration of potential toxic elements and the pollution indices.

	As	Cd	Co	Cr	Cu	Mn	Ni	Pb	Sb	Zn	HPI	HEI	NP
As	1												
Cd	.818^a^	1											
Co	.764^a^	.796^a^	1										
Cr	.637^a^	.792^a^	.439^b^	1									
Cu	.781^a^	.939^a^	.688^a^	.819^a^	1								
Mn	.492^b^	.649^a^	.824^a^	.408	.540^b^	1							
Ni	.663^a^	.816^a^	.910^a^	.404	.695^a^	.757^a^	1						
Pb	.838^a^	.969^a^	.739^a^	.865^a^	.927^a^	.615^a^	.688^a^	1					
Sb	−.280	−.184	−.584^a^	.286	−.030	−.453^b^	−.658^a^	−.024	1				
Zn	.790^a^	.970^a^	.834^a^	.699^a^	.927^a^	.684^a^	.902^a^	.902^a^	−.327	1			
HPI	.766^a^	.930^a^	.750^a^	.828^a^	.896^a^	.649^a^	.732^a^	.934^a^	−.085	.895^a^	1		
HEI	.752^a^	.921^a^	.850^a^	.758^a^	.848^a^	.867^a^	.790^a^	.924^a^	−.210	.896^a^	.904^a^	1	
NP	.510^b^	.671^a^	.845^a^	.426	.563^a^	.998^a^	.775^a^	.635^a^	−.451^b^	.705^a^	.668^a^	.879^a^	1

a Correlation is significant at the .01 level (2-tailed).

b Correlation is significant at the .05 level (2-tailed).

The Cr concentrations were positively correlated with Cu and Pb. The Cu concentrations had a relatively high significant positive correlation with Cd, Pb, and Zn. Copper (Cu) concentrations often exhibit a significant positive correlation with cadmium (Cd), lead (Pb), and zinc (Zn) in contaminated surface waters due to their shared industrial origins and similar geochemical behavior. These metals are frequently released together from activities such as metal plating, mining, and industrial wastewater discharge [71]. Their tendency to bind to similar particles and undergo comparable processes of transportation and deposition in aquatic environments further explains their co-occurrence in polluted water systems [72].

The Mn was only correlated with Co. The Ni concentrations were positively correlated with Co and Zn. The Pb concentrations had a significant positive correlation with Cu, Cd, and Zn. The Zn concentrations showed a highly significant positive correlation with Pb, Ni, Cu, Co, and Cd. The r values greater than .90 were observed between Cd-Cu, Pb, and Zn; Co-Ni, Cu-Pb, and Zn; Ni-Zn; and Pb-Zn [72]. The strong, significant positive correlations among Cd, Co, Cr, Cu, Ni, Pb, and Zn suggested similar sources in the study area [71]. In contrast, As and Mn are probably derived from the bottom sediments through reduction processes in the presence of microbial activities. Antimony (Sb) often shows a good correlation with cobalt (Co), manganese (Mn), and nickel (Ni) in surface water in textile industrial areas due to shared sources and geochemical behavior (Table 4). These metals are commonly used in industrial processes, including textile dyeing, metal finishing, and wastewater discharge, leading to their co-release into the environment [73]. Additionally, the adsorption and mobility of these metals in water are influenced by similar physicochemical conditions, such as pH and redox potential, resulting in their co-occurrence and similar distribution patterns in polluted water bodies [74].

Subsequently, this study revealed that antimony's (Sb) has strong correlation with cobalt (*r* = .584, *α* = 0.95), manganese (*r* = .453, *α* = 0.95), nickel (.658, *α* = 0.95), which are presented in Table 4. It might be happened due to the reasons that antimony (Sb) is often associated with elements like cobalt (Co), manganese (Mn), and nickel (Ni) in various environmental and geological contexts, reflecting their shared geochemical behavior and occurrence in sulfide and oxide minerals. For instance, studies have identified correlations between Sb and Co in mining areas, suggesting their co-occurrence in ore deposits and potential simultaneous mobilization during mining activities [75]. Similarly, Sb and Mn frequently co-occur in sedimentary and aqueous environments, where manganese oxides act as scavengers for antimony, influencing its speciation and mobility [76]. In the case of Ni, the correlation is observed in metallurgical processes and natural deposits, where both elements are enriched in ultramafic rocks and related hydrothermal systems. These relationships highlight the need for integrated studies on Sb's behavior alongside these metals to better understand their environmental dynamics and risks in contaminated sites.

### 3.5 Assessment of potential ecological risk index (PERI)

**Fig. 6:**
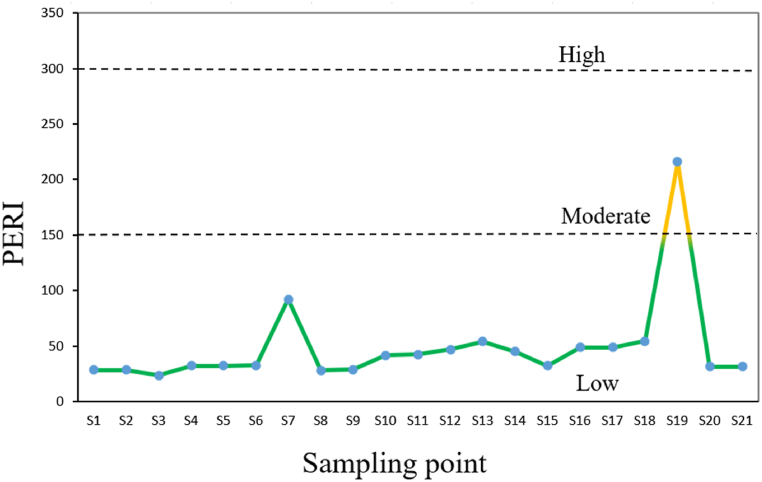
Potential ecological risk index of PTEs at various sampling point of Mokosh Beel.

This study has a special focus on the ecological consequences of PTEs on aquatic organisms. This is because potentially toxic elements (PTEs) have become an increasing concern in aquatic ecosystems due to their persistence, bioaccumulation, and potential harm to aquatic life and humans [77]. Unlike organic pollutants, PTEs such as arsenic (As), cadmium (Cd), lead (Pb), and mercury (Hg) do not degrade over time, leading to long-term contamination. They can accumulate in the tissues of aquatic organisms, entering the food chain and posing risks to human health through consumption [78,79]. Prolonged exposure to these elements can result in toxic effects, including disruptions to metabolic and reproductive functions in aquatic species [80]. The probable ecological impacts on aquatic living organisms were computed in this study in terms of the potential ecological risk index (PERI), and the results are displayed in Fig. 6. The findings revealed that almost all the sampling points in Mokosh Beel were under the low degree of risk level; only sampling point S-19 was the exception, where the PERI value was much greater than 150. Thus, the pollution monitoring of the ecological risk of Mokosh Beel water imposed by these PTEs is not a major concern. Although the findings revealed a low concern for the overall ecological risk levels, the elevated ecological risk factor of certain PTEs remains a matter of concern, as these metals can bioaccumulate in the tissues of organisms over time and have adverse effects on respiratory, gastrointestinal, and animal metabolism and overall population dynamics [80]. For instance, Cr and Cd accumulation in fish can result in disturbances in growth and reproduction, as well as histopathological alterations in vital organs. These adverse effects can disrupt the overall population dynamics of aquatic species, emphasizing the need for ongoing monitoring and mitigation strategies to address the specific risks posed by Cd and Cr in Mokosh Beel.

### 3.6 Non-carcinogenic and carcinogenic health risk assessment

The health risk assessment determines the levels of exposure to a contaminant in the human body and provides a quantitative risk to human health [54]. In the present study, probable non-carcinogenic and carcinogenic health risks were estimated for direct oral and dermal pathways. Non-carcinogenic health risks were assessed by calculating the Hazard Quotient (HQ) and Hazard Index (HI), whereas the possible cancer risks were estimated based on the Carcinogenic Risk (CR) for both adults and children.

**Fig. 7:**
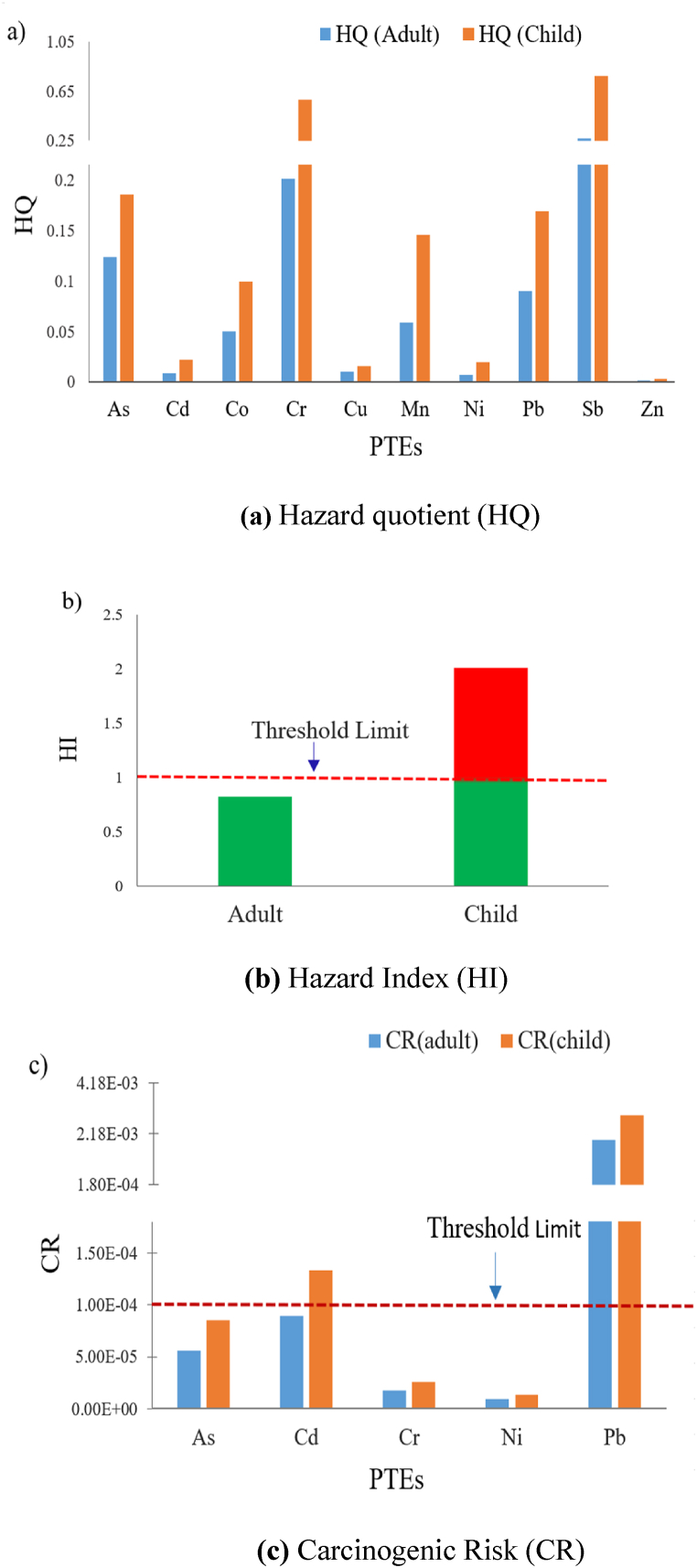
(a) Hazard quotient (HQ), **(b)** hazard Index (HI), and **(c)** carcinogenic risk (CR) of different potentially toxic elements for both adults and children.

Potential non-carcinogenic health risks from exposure to ten PTEs, viz., Cd, Cr, As, Co, Ni, Cu, Mn, Pb, Zn, and Sb, are displayed in Fig. 7a. The results showed that HQ values were less than the threshold limit (HQ = 1) for all these metals, indicating little hazard was posed by these metals. However, the HQ values for children in the cases of Cr (HQ = .58) and Sb (HQ = .77) were very close to the threshold limit. This proximity suggests a potential health risk to children associated with these contaminants. The exposure to Cr and Sb imposed higher potential health risks through dermal contact rather than direct oral ingestion. The potential non-carcinogenic health hazards associated with Cr include lung and nasal ulcers, bronchial asthma, skin allergies, and reproductive and developmental problems; in extreme cases, excess exposure to Cr may cause death [81]. Sb at low concentration causes irritation in the eyes, skin, and lungs, but prolonged exposure to Sb can cause electrocardiogram changes, diarrhea, stomachache, emesis, and gastric ulcers. However, the HQ values for the rest of the PTEs were significantly lower than the threshold limit, indicating that these metals are relatively free from non-carcinogenic health hazards. Overall, the degrees of the non-carcinogenic hazard quotient followed a decreasing order of Sb > Cr > As > Pb > Mn > Co > Cd > Cu > Ni > Zn. Again, the non-carcinogenic hazard index (HI) values for children and adults were found to be 2.012 and .823, respectively (Fig. 7b). This indicates children are the main victims of the probable non-carcinogenic risk imposed by these toxic metals. In another study, Moniruzzaman et al. (2020) investigated the probable human health risks associated with different aquifers in the vicinity of our study area and disclosed that HI values for both the adults and children exceeded 1. This included, particularly in the midway and shallow groundwater, indicating significant non-cancer risks endured in the floodplain area. Toxic metals, particularly As, Cr, and Pb, were the major contributors to inducing those potential health hazards. Yazman et al. [82] explored the implications for public health associated with toxic elements in the groundwater of the Southern Coast of the Black Sea in Türkiye, in which the THQ values for both the adults (.07) and children (.08) were far below the threshold limit. This implies no non-carcinogenic health risks exist in all the sampling stations. Moreover, Karadeniz et al. [57] revealed that the HQ and HI values of all the sampling points in the plateaus of Giresun Province of Türkiye were below the safety limit, ensuring the suitability of the plateau water for domestic purposes without posing any non-carcinogenic health risks.

Potential carcinogenic health risks were evaluated for Cr, As, Ni, Cd, and Pb, which are depicted in Fig. 7c. The results show that CR values of Pb for both adults (1.94 × 10⁻³) and children (2.90 × 10⁻³) surpassed the carcinogenic critical limit of 1 × 10⁻⁴, indicating that it can cause serious cancer risk not only for children but also for adults. Probable cancer risk that might be imposed by Pb includes renal autism, arthritis, dysfunction, and other birth defects [33]. The calculated carcinogenic risk is also higher for Cd, in which CR values for children (1.30 × 10⁻⁴) exceeded the threshold limit and for adults (8.94 × 10⁻⁵) were nearly close to the threshold limit. Possible cancer risk due to exposure to Cd particularly includes lung, jugular, prostate, and pancreatic cancer. Long-term exposure to Cd may also cause breast cancer. However, the potential cancer risk associated with As is also a matter of concern, as its CR values are highly close to the threshold limit for both adults (5.68 × 10⁻⁵) and children (8.48 × 10⁻⁵). Arsenic imposes serious potential cancer risk even at low concentrations, which might be responsible for skin and lung cancer and also some chronic toxicity, including keratosis and pigmentation [33]. According to Fig. 7c, the potential carcinogenic health risk of the measured parameters followed a decreasing order of Pb > Cd > As > Cr > Ni. Moniruzzaman et al. (2024) also explored the carcinogenic human health risks associated with various aquifers located near our study region, in which the CR values of As, Cr, and Ni exceeded the threshold limit, indicating a momentous probability of inducing cancer. Yazman et al. [82] reported that the CR values for arsenic in the groundwater of the Southern Coast of the Black Sea in Türkiye exceeded the international safety limit, and also from Tokatli et al. [83], arsenic had the highest carcinogenic risks. Therefore, arsenic is the dominant toxic element in inducing cancer that underscores the urgency of oppressive monitoring and research to diminish the exposure and safeguard public health.

Overall, in both cases, the health risk assessment suggests that children are in a more vulnerable state compared to adults concerning PTEs. This vulnerability highlights the importance of taking special precautions and preventive measures to protect children from potential health risks associated with water pollution.

## 4 Conclusion

This study comprehensively assessed the surface water quality of Mokosh Beel by analyzing the concentrations of potentially toxic elements (PTEs) and physicochemical parameters. The results demonstrated that most water quality parameters, except pH, exceeded both local and international guideline thresholds, highlighting significant water quality deterioration. Among the PTEs, Mn, Pb, and Cr were identified as the primary pollutants, with Mn and Pb showing the highest pollution levels according to the single-factor pollution index. The Nemerow pollution index indicated mild to moderate pollution, while both the heavy metal pollution index (HPI) and the heavy metal evaluation index (HEI) suggested only slight pollution across most sampling sites.

Principal component analysis (PCA) and hierarchical cluster analysis (HCA) identified industrial effluent, particularly from tannery, leather, and paint industries, as the main source of PTE contamination. These findings are critical for understanding the extent of industrial pollution affecting the Beel. In terms of human health risks, children were found to be at higher non-carcinogenic risk due to PTE exposure, while both children and adults face significant carcinogenic risks, particularly from Pb and Cd. Encouragingly, arsenic (As), a known carcinogen, did not pose a substantial health risk in this study, which is a positive outcome for the region.

From an ecological standpoint, the potential ecological risk index (PERI) indicated that PTEs in Mokosh Beel do not pose a significant threat to aquatic organisms, although the long-term bioaccumulation potential of certain elements like Cd and Cr remains a concern. This study underscores the critical need for targeted pollution mitigation strategies to address industrial sources of contamination and protect both human and ecological health in the region. The findings serve as an urgent call for policy interventions and sustainable water management practices to safeguard Mokosh Beel from further degradation. A detailed ecotoxicological study involving laboratory or field experiments would provide additional insights into the biological impact of these metals, and that could be pursued as individual research in the future. Future studies could be expanded on this work by incorporating bioassays or in situ toxicity studies to validate and complement the risk estimates derived from metal concentrations.

## CRediT authorship contribution statement

**Md. Shahriar Mahmud:** Writing – review & editing, Writing – original draft, Methodology, Data curation. **M. Safiur Rahman:** Writing – review & editing, Supervision. **S.A. Dina:** Formal analysis, Data curation. **M. Rifat Nasher:** Writing – review & editing, Visualization. **Tasrina R. Choudhury:** Visualization. **Bilkis A. Begum:** Visualization. **Abdus Samad:** Writing – review & editing, Visualization, Supervision, Investigation, Conceptualization.

## Data availability

The data associated with this paper has been provided in the supplementary materials.

## Declaration of competing interest

The authors declare no conflict of interest.
