# Supplementary material for: Potential toxic elements in surface water of Mokosh Beel, Gazipur, Bangladesh: Ecological and human health risk assessment for recreational users
Source: Heliyon. 2025 Jan 31;11(3):e42421. doi: 10.1016/j.heliyon.2025.e42421 (PMC11847065; doi:10.1016/j.heliyon.2025.e42421)
Supplement: Multimedia component 1 [file mmc1.docx]

Potential toxic elements in surface water of Mokosh Beel, Gazipur, Bangladesh: Ecological and human health risk assessment for recreational users

M. Shahriar Mahmud^1^, M. Safiur Rahman^2*^_,_ S.A. Dina^1^, M. Rifat Nasher^3^, Tasrina R. Choudhury^2^, Bilkis A. Begum^2^ and, Abdus Samad^1,*^

^1^Department of Chemistry, Jagannath University, Dhaka-1100, Bangladesh

^2^Water Quality Research Laboratory, Chemistry Division, Water & Air Research Cell (WARC), Atomic Energy Center, Bangladesh Atomic Energy Commission, Dhaka-1000, Bangladesh

^3^Department of Geography and Environment, Jagannath University, Dhaka-1100, Bangladesh

Table S1: GPS locations of sampling points.

| Sampling ID | Longitude | Lattitude |
| --- | --- | --- |
| SC -1 | 24.081595 | 90.286194 |
| SC-2 | 24.079516 | 90.286496 |
| SC -3 | 24.0775494 | 90.2885232 |
| SC -4 | 24.0886219 | 90.2797219 |
| SC -5 | 24.0858739 | 90.2769136 |
| SC -6 | 24.0837730 | 90.2734281 |
| SC -7 | 24.0814953 | 90.2743588 |
| SC -8 | 24.0788766 | 90.2737439 |
| SC -9 | 24.0802014 | 90.2725802 |
| SC -10 | 24.0824485 | 90.2702101 |
| SC -11 | 24.0839373 | 90.2696854 |
| SC -12 | 24.0837928 | 90.2682635 |
| SC -13 | 24.0857827 | 90.2675034 |
| SC -14 | 24.0876198 | 90.2664406 |
| SC -15 | 24.0876339 | 90.2687319 |
| SC -16 | 24.0876269 | 90.2703717 |
| SC -17 | 24.0859737 | 90.2727156 |
| SC -18 | 24.0869171 | 90.2746195 |
| SC -19 | 24.0863214 | 90.2761636 |
| SC -20 | 24.0857947 | 90.2787690 |
| SC -21 | 24.0871788 | 90.2786808 |

**Table S2.** Physicochemical water quality parameters of surface water samples collected from Mokosh Beel.

| Sample Code | pH | DO (mg/L) | EC (μS/cm) | Salinity (mg/L) | TDS (mg/L) |
| --- | --- | --- | --- | --- | --- |
| SC -1 | 7.44 | 6.19 | 2790 | 1400 | 1396 |
| SC-2 | 7.28 | 4.24 | 2990 | 1520 | 1496 |
| SC -3 | 7.79 | 4.99 | 2050 | 990 | 1020 |
| SC -4 | 7.19 | 5.73 | 3040 | 1540 | 1520 |
| SC -5 | 7.41 | 4.61 | 3180 | 1620 | 1590 |
| SC -6 | 7.49 | 2.20 | 3220 | 1740 | 1610 |
| SC -7 | 7.36 | 1.43 | 2885 | 1550 | 1530 |
| SC -8 | 7.71 | 2.38 | 3120 | 1580 | 1560 |
| SC -9 | 7.66 | 3.67 | 2772 | 1390 | 1381 |
| SC -10 | 7.33 | 2.73 | 2810 | 1430 | 1415 |
| SC -11 | 7.74 | 2.22 | 3410 | 1750 | 1700 |
| SC -12 | 7.74 | 2.26 | 3310 | 1690 | 1650 |
| SC -13 | 7.85 | 2.67 | 3270 | 1670 | 1640 |
| SC -14 | 7.70 | 2.56 | 3380 | 1730 | 1690 |
| SC -15 | 7.66 | 3.43 | 3330 | 1700 | 1660 |
| SC -16 | 7.81 | 4.28 | 3340 | 1710 | 1670 |
| SC -17 | 7.79 | 3.77 | 3290 | 1680 | 1640 |
| SC -18 | 7.65 | 3.48 | 3240 | 1650 | 1620 |
| SC -19 | 7.52 | 4.88 | 3220 | 1640 | 1610 |
| SC -20 | 7.62 | 4.07 | 3120 | 1580 | 1560 |
| SC -21 | 7.66 | 4.73 | 3100 | 1570 | 1550 |
| Mean | 7.59 | 3.69 | 3088.905 | 1577.619 | 1548 |
| Median | 7.66 | 3.67 | 3180 | 1620 | 1590 |
| Standard Deviation | 0.191454 | 1.315 | 308.166 | 172.3051 | 152.3539 |
| Variance | 0.0367 | 1.730 | 162975.7 | 29689.05 | 23211.7 |
| Minimum | 7.19 | 1.43 | 2050 | 1750 | 1020 |
| Maximum | 7.85 | 6.19 | 3410 | 33130 | 1700 |

**Table S3.** The concentration of potential toxic elements in surface water of Mokosh Beel and the results of pollution indices.

| Sample Code | Concentration of potential toxic elements (µg/L) | | | | | | | | | |  | Pollution indices | | |
| --- | --- | --- | --- | --- | --- | --- | --- | --- | --- | --- | --- | --- | --- | --- |
|  | **As** | **Cd** | **Co** | **Cr** | **Cu** | **Mn** | **Ni** | **Pb** | **Sb** | **Zn** |  | **HPI** | **HEI** | **N_P_** |
| SC -1 | 2.60 | 0.53 | 0.75 | 25.23 | 21.38 | 93.85 | 8.68 | 37.42 | 11.03 | 61.97 |  | 24.99 | 3.03 | 0.70 |
| SC-2 | 2.32 | 0.59 | 0.60 | 22.96 | 14.06 | 166.15 | 5.70 | 43.12 | 11.16 | 25.68 |  | 28.17 | 3.79 | 1.20 |
| SC -3 | 0.23 | 0.54 | 7.98 | 12.24 | 1.32 | 571.03 | 27.01 | 1.02 | 2.51 | 76.24 |  | 27.48 | 6.58 | 4.06 |
| SC -4 | 0.12 | 0.69 | 0.54 | 30.13 | 21.68 | 177.81 | 6.08 | 34.69 | 16.35 | 28.12 |  | 33.13 | 4.12 | 1.29 |
| SC -5 | 2.06 | 0.66 | 0.55 | 31.12 | 21.70 | 65.15 | 5.54 | 37.45 | 12.78 | 28.49 |  | 27.51 | 2.92 | 0.57 |
| SC -6 | 1.78 | 0.58 | 0.79 | 32.14 | 25.67 | 306.36 | 7.48 | 47.22 | 15.95 | 73.16 |  | 37.19 | 5.72 | 2.20 |
| SC -7 | 0.25 | 2.13 | 0.79 | 38.75 | 42.03 | 282.87 | 24.49 | 101.78 | 12.08 | 312.29 |  | 59.40 | 7.03 | 2.06 |
| SC -8 | 0.10 | 0.58 | 0.72 | 24.15 | 26.96 | 282.58 | 4.74 | 31.47 | 13.40 | 33.67 |  | 31.63 | 4.81 | 2.06 |
| SC -9 | 0.11 | 0.59 | 0.58 | 23.73 | 23.72 | 294.62 | 3.82 | 37.98 | 16.03 | 38.67 |  | 34.72 | 5.18 | 2.12 |
| SC -10 | 0.95 | 0.91 | 0.74 | 29.37 | 20.58 | 295.49 | 4.65 | 66.18 | 14.88 | 44.42 |  | 4.65 | 5.89 | 2.13 |
| SC -11 | 0.10 | 0.86 | 0.59 | 37.14 | 23.16 | 284.72 | 4.27 | 79.37 | 18.50 | 43.19 |  | 45.77 | 6.35 | 2.06 |
| SC -12 | 1.68 | 0.97 | 0.71 | 39.42 | 28.27 | 295.66 | 4.13 | 69.51 | 15.01 | 45.38 |  | 44.20 | 6.20 | 2.14 |
| SC -13 | 1.76 | 1.11 | 0.77 | 46.02 | 23.06 | 302.96 | 5.47 | 91.85 | 16.39 | 71.47 |  | 50.47 | 6.96 | 2.20 |
| SC -14 | 0.10 | 0.95 | 0.50 | 38.42 | 22.62 | 296.85 | 4.36 | 88.56 | 16.98 | 41.87 |  | 47.36 | 6.63 | 2.15 |
| SC -15 | 0.10 | 0.59 | 0.77 | 38.56 | 21.85 | 306.98 | 4.51 | 48.61 | 14.73 | 32.01 |  | 37.14 | 5.75 | 2.21 |
| SC -16 | 1.49 | 1.02 | 0.69 | 37.64 | 21.88 | 297.72 | 4.45 | 84.33 | 15.64 | 60.37 |  | 46.76 | 6.52 | 2.16 |
| SC -17 | 0.09 | 1.08 | 0.58 | 31.92 | 23.77 | 193.60 | 4.10 | 87.84 | 17.28 | 50.88 |  | 45.23 | 5.49 | 1.42 |
| SC -18 | 3.16 | 1.24 | 0.67 | 32.60 | 18.94 | 197.86 | 5.33 | 92.36 | 15.44 | 47.57 |  | 46.80 | 5.65 | 1.45 |
| SC -19 | 9.73 | 4.81 | 15.93 | 64.70 | 91.01 | 724.30 | 45.01 | 341.29 | 10.11 | 671.79 |  | 135.52 | 17.86 | 5.27 |
| SC -20 | 0.59 | 0.65 | 0.62 | 31.03 | 20.33 | 180.14 | 5.23 | 40.30 | 14.01 | 21.92 |  | 31.81 | 4.15 | 1.31 |
| SC -21 | 0.46 | 0.66 | 0.89 | 30.01 | 20.34 | 35.04 | 5.67 | 38.53 | 16.27 | 30.60 |  | 29.00 | 2.77 | 0.61 |
| Mean | 1.43 | 1.04 | 1.79 | 33.25 | 25.44 | 269.13 | 9.08 | 71.47 | 14.12 | 87.61 |  | 43.14 | 5.88 | 1.95 |
| Maximum | 9.73 | 4.80 | 15.93 | 64.70 | 91.00 | 724.30 | 45.01 | 341.29 | 18.49 | 671.79 |  | 135.52 | 17.86 | 5.27 |
| Minimum | 0.09 | 0.53 | 0.50 | 12.24 | 1.32 | 35.04 | 3.82 | 1.02 | 2.51 | 21.91 |  | 4.65 | 2.77 | 0.57 |
| GM | 0.56 | 0.86 | 0.87 | 31.68 | 21.23 | 225.74 | 6.62 | 50.71 | 13.35 | 52.95 |  | - | - | - |
| CV% | 149.4 | 90.6 | 202.0 | 31.1 | 65.2 | 57.0 | 114.0 | 94.2 | 24.6 | 167.6 |  | - | - | - |

| **Table S4.** Variation of single factor pollution index for each element with their mean values at various sampling points.   \| Code \| SC-1 \| SC-2 \| SC-3 \| SC-4 \| SC-5 \| SC-6 \| SC-7 \| SC-8 \| SC-9 \| SC-10 \| SC-11 \| \| --- \| --- \| --- \| --- \| --- \| --- \| --- \| --- \| --- \| --- \| --- \| --- \| \| P_i_(As) \| 0.051 \| 0.046 \| 0.004 \| 0.002 \| 0.041 \| 0.035 \| 0.005 \| 0.002 \| 0.002 \| 0.019 \| 0.002 \| \| P_i_(Cd) \| 0.106 \| 0.118 \| 0.107 \| 0.1386 \| 0.132 \| 0.115 \| 0.426 \| 0.426 \| 0.118 \| 0.181 \| 0.171 \| \| P_i_(Co) \| 0.007 \| 0.006 \| 0.079 \| 0.005 \| 0.005 \| 0.007 \| 0.007 \| 0.007 \| 0.005 \| 0.007 \| 0.005 \| \| P_i_(Cr) \| 0.504 \| 0.459 \| 0.244 \| 0.602 \| 0.622 \| 0.642 \| 0.775 \| 0.775 \| 0.474 \| 0.587 \| 0.742 \| \| P_i_(Cu) \| 0.021 \| 0.014 \| 0.001 \| 0.021 \| 0.021 \| 0.025 \| 0.042 \| 0.042 \| 0.023 \| 0.020 \| 0.023 \| \| P_i_(Mn) \| 0.938 \| 1.661 \| 5.710 \| 1.778 \| 0.651 \| 3.063 \| 2.822 \| 2.828 \| 2.946 \| 2.954 \| 2.847 \| \| P_i_(Ni) \| 0.086 \| 0.0569 \| 0.270 \| 0.060 \| 0.055 \| 0.074 \| 0.244 \| 0.244 \| 0.038 \| 0.046 \| 0.042 \| \| P_i_(Pb) \| 0.748 \| 0.862 \| 0.020 \| 0.693 \| 0.749 \| 0.944 \| 2.035 \| 2.035 \| 0.759 \| 1.323 \| 1.587 \| \| P_i_(Sb) \| 0.551 \| 0.557 \| 0.125 \| 0.817 \| 0.638 \| 0.797 \| 0.604 \| 0.604 \| 0.801 \| 0.744 \| 0.924 \| \| P_i_(Zn) \| 0.012 \| 0.005 \| 0.015 \| 0.005 \| 0.005 \| 0.014 \| 0.062 \| 0.062 \| 0.007 \| 0.008 \| 0.008 \|  \| Code \| SC-12 \| SC-13 \| SC-14 \| SC-15 \| SC-16 \| SC-17 \| SC-18 \| SC-19 \| SC-20 \| SC-21 \| Mean (P_i_) \| \| --- \| --- \| --- \| --- \| --- \| --- \| --- \| --- \| --- \| --- \| --- \| --- \| \| P_i_(As) \| 0.033 \| 0.035 \| 0.002 \| 0.002 \| 0.029 \| 0.001 \| 0.063 \| 0.194 \| 0.011 \| 0.009 \| 0.028 \| \| P_i_(Cd) \| 0.194 \| 0.222 \| 0.190 \| 0.118 \| 0.203 \| 0.216 \| 0.247 \| 0.961 \| 0.130 \| 0.132 \| 0.207 \| \| P_i_(Co) \| 0.007 \| 0.007 \| 0.004 \| 0.007 \| 0.006 \| 0.005 \| 0.006 \| 0.159 \| 0.006 \| 0.008 \| 0.017 \| \| P_i_(Cr) \| 0.788 \| 0.920 \| 0.768 \| 0.771 \| 0.752 \| 0.638 \| 0.651 \| 1.294 \| 0.620 \| 0.600 \| 0.664 \| \| P_i_(Cu) \| 0.028 \| 0.023 \| 0.022 \| 0.021 \| 0.021 \| 0.023 \| 0.018 \| 0.091 \| 0.020 \| 0.020 \| 0.025 \| \| P_i_(Mn) \| 2.956 \| 3.029 \| 2.968 \| 3.069 \| 2.977 \| 1.935 \| 1.978 \| 7.243 \| 1.801 \| 0.350 \| 2.691 \| \| P_i_(Ni) \| 0.041 \| 0.054 \| 0.043 \| 0.045 \| 0.044 \| 0.041 \| 0.053 \| 0.450 \| 0.052 \| 0.056 \| 0.090 \| \| P_i_(Pb) \| 1.390 \| 1.836 \| 1.771 \| 0.972 \| 1.688 \| 1.758 \| 1.847 \| 6.825 \| 0.806 \| 0.770 \| 1.429 \| \| P_i_(Sb) \| 0.750 \| 0.819 \| 0.848 \| 0.736 \| 0.781 \| 0.864 \| 0.771 \| 0.505 \| 0.700 \| 0.813 \| 0.705 \| \| P_i_(Zn) \| 0.009 \| 0.014 \| 0.008 \| 0.006 \| 0.012 \| 0.010 \| 0.009 \| 0.134 \| 0.004 \| 0.006 \| 0.017 \|   **Table S5. Rotated Component Matrix^a^** | | |
| --- | --- | --- | --- | --- | --- | --- | --- | --- | --- | --- | --- | --- | --- | --- | --- | --- | --- | --- | --- | --- | --- | --- | --- | --- | --- | --- | --- | --- | --- | --- | --- | --- | --- | --- | --- | --- | --- | --- | --- | --- | --- | --- | --- | --- | --- | --- | --- | --- | --- | --- | --- | --- | --- | --- | --- | --- | --- | --- | --- | --- | --- | --- | --- | --- | --- | --- | --- | --- | --- | --- | --- | --- | --- | --- | --- | --- | --- | --- | --- | --- | --- | --- | --- | --- | --- | --- | --- | --- | --- | --- | --- | --- | --- | --- | --- | --- | --- | --- | --- | --- | --- | --- | --- | --- | --- | --- | --- | --- | --- | --- | --- | --- | --- | --- | --- | --- | --- | --- | --- | --- | --- | --- | --- | --- | --- | --- | --- | --- | --- | --- | --- | --- | --- | --- | --- | --- | --- | --- | --- | --- | --- | --- | --- | --- | --- | --- | --- | --- | --- | --- | --- | --- | --- | --- | --- | --- | --- | --- | --- | --- | --- | --- | --- | --- | --- | --- | --- | --- | --- | --- | --- | --- | --- | --- | --- | --- | --- | --- | --- | --- | --- | --- | --- | --- | --- | --- | --- | --- | --- | --- | --- | --- | --- | --- | --- | --- | --- | --- | --- | --- | --- | --- | --- | --- | --- | --- | --- | --- | --- | --- | --- | --- | --- | --- | --- | --- | --- | --- | --- | --- | --- | --- | --- | --- | --- | --- | --- | --- | --- | --- | --- | --- | --- | --- | --- | --- | --- | --- | --- | --- | --- | --- | --- | --- | --- | --- | --- | --- | --- | --- | --- | --- | --- | --- | --- | --- | --- | --- | --- | --- | --- | --- | --- | --- | --- | --- |
|  | Components | |
|  | PC1 | PC2 |
| As | **0.757** | 0.407 |
| Cd | **0.903** | 0.394 |
| Co | 0.560 | **0.785** |
| Cr | **0.946** | -0.111 |
| Cu | **0.934** | 0.235 |
| Mn | 0.454 | **0.691** |
| Ni | 0.523 | **0.818** |
| Pb | **0.956** | 0.248 |
| Sb | 0.214 | **-0.940** |
| Zn | **0.822** | 0.525 |
| Eigenvalues | **7.146** | **1.811** |
| % of variances | **71.46** | **18.11** |
| Cumulative % of variances | **71.46** | **89.56** |
| Extraction Method: Principal Component Analysis.  Rotation Method: Varimax with Kaiser Normalization. | | |
| 1. Rotation converged in 3 iterations.   **Table S6: Levels of Non-carcinogenic risk for adult and child.** | | |

| PTEs | HQ_ingestion_ |  | HQ_dermal_ |  | Hazard Quotient (HQ) |  |
| --- | --- | --- | --- | --- | --- | --- |
|  | **Adult** | **Child** | **Adult** | **Child** | **Adult** | **Child** |
| As | 0.1230 | 0.1837 | 0.0007 | 0.0020 | 0.1237 | 0.1858 |
| Cd | 0.0028 | 0.0042 | 0.0059 | 0.0174 | 0.0087 | 0.0217 |
| Co | 0.0327 | 0.0488 | 0.0170 | 0.0504 | 0.0498 | 0.0993 |
| Cr | 0.0115 | 0.0172 | 0.1899 | 0.5603 | 0.2014 | 0.5775 |
| Cu | 0.0099 | 0.0148 | 0.0003 | 0.0008 | 0.0102 | 0.0157 |
| Mn | 0.0184 | 0.0275 | 0.0400 | 0.1182 | 0.0585 | 0.1458 |
| Ni | 0.0004 | 0.0007 | 0.0064 | 0.0191 | 0.0069 | 0.0199 |
| Pb | 0.0654 | 0.0977 | 0.0243 | 0.0717 | 0.0897 | 0.1695 |
| Sb | 0.0193 | 0.0288 | 0.2523 | 0.7446 | 0.2717 | 0.7734 |
| Zn | 0.0016 | 0.0023 | 0.0001 | 0.0003 | 0.0017 | 0.0027 |
| Hazard Index(HI) |  |  |  |  | **0.82280** | **2.01165** |

**Table S7:** Carcinogenic risk for adult and child.

| Heavy Metal | CR_ingestion_ |  | CR_dermal_ |  | Carcinogenic Risk (CR) | |
| --- | --- | --- | --- | --- | --- | --- |
|  | **Adult** | **Child** | **Adult** | **Child** | **Adult** | **Child** |
| As | 5.536E-05 | 8.267E-05 | 7.422E-07 | 2.189E-06 | 5.610E-05 | 8.486E-05 |
| Cd | 8.939E-05 | 0.00013 | - | - | 8.939E-05 | 0.00013 |
| Cr | 1.728E-05 | 2.581E-05 | - | - | 1.728E-05 | 2.581E-05 |
| Ni | 9.056E-06 | 1.352E-05 | - | - | 9.056E-06 | 1.352E-05 |
| Pb | 0.00195 | 0.00290 | - | - | 0.00194 | 0.00290 |


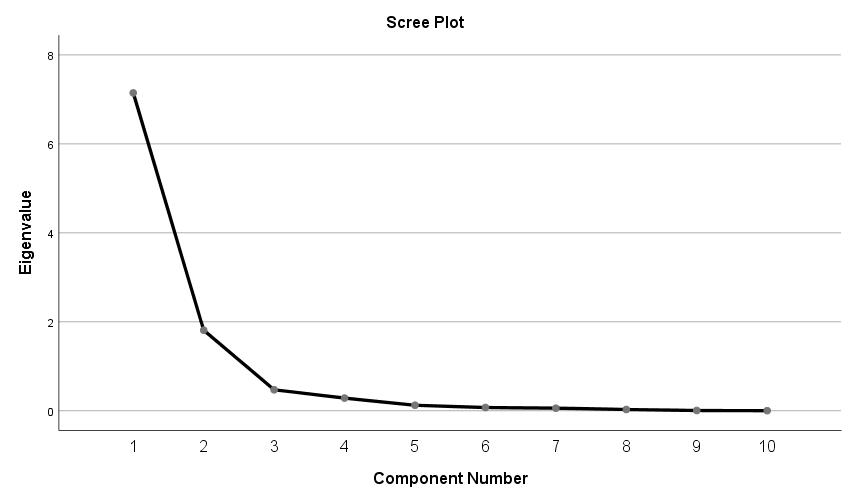


**Fig. S1**: Scree plot of the characteristics root (eigen value) with component factor.
